# Graphite furnace atomic absorption spectrometry as a routine method for the quantification of beryllium in blood and serum

**DOI:** 10.1186/1752-153X-2-14

**Published:** 2008-07-02

**Authors:** Chadi H Stephan, Michel Fournier, Pauline Brousseau, Sébastien Sauvé

**Affiliations:** 1 Department of Chemistry, Université de Montréal, P.O. 6128, Station Centre-Ville, Montréal, QC, Canada, H3C 3J7; 2 INRS-Institut Armand-Frappier, 245 Hymus, Pointe-Claire, QC, Canada H9R 3G6

## Abstract

**Background:**

A routine method for the quantification of beryllium in biological fluids is essential for the development of a chelation therapy for Chronic Beryllium Disease (CBD). We describe a procedure for the direct determination of beryllium in undigested micro quantities of human blood and serum using graphite furnace atomic absorption spectrometry. Blood and serum samples are prepared respectively by a simple 8-fold and 5-fold dilution with a Nash Reagent. Three experimental setups are compared: using no modifier, using magnesium nitrate and using palladium/citric acid as chemical modifiers.

**Results:**

In serum, both modifiers did not improve the method sensitivity, the optimal pyrolysis and atomization temperatures are 1000°C and 2900°C, respectively. In blood, 6 μg of magnesium nitrate was found to improve the method sensitivity. The optimal pyrolysis and atomization temperatures were 800°C and 2800°C respectively.

**Conclusion:**

In serum, the method detection limit was 2 ng l^-1^, the characteristic mass was 0.22 (± 0.07) pg and the accuracy ranged from 95 to 100%. In blood, the detection limit was 7 ng l^-1^, the characteristic mass was 0.20 (± 0.02) pg and the accuracy ranged from 99 to 101%.

## Background

Beryllium is the 35^th ^most abundant element in the earth's crust, with an average of 6 mg kg^-1 ^[1]. It has unique physical and chemical properties that improves the characteristics of alloys producing greater tensile strength, high electrical and thermal conductivity, along with good corrosion and fatigue resistance [2]. Beryllium and its metal alloys have been widely used for electrical equipment, electronic instrumentation, structural components for aircraft, missiles, satellites and nuclear reactors [3,4].

Beryllium and its compounds are very toxic. They can cause chronic beryllium disease (CBD), a lung disorder initiated by an electrostatic interaction with the MHC class II human leukocyte antigen (HLA) [5]. Recent molecular epidemiological studies found a significant correlation between the risk of developing CBD and the predicted surface electrostatic potential of the HLA-DP alleles, suggesting that individuals who carry the most negatively charged alleles are at greater risk of beryllium sensitization and CBD [6]. Because of these findings, increased research efforts are being targeted towards the development of a CBD treatment by chelation therapy [7,8]. For that purpose, a routine method is required to analyse beryllium in micro samples of biological fluids with high sensitivity, accuracy, and low detection limits.

The commonly used methods for the determination of beryllium in tissues and urine include fluorometric [9,10], gas chromatographic [11-14], atomic emission [15-17], inductively coupled plasma mass spectrometry (ICP-MS) [18-21] and atomic absorption [22-25]. Fluorometric methods are sensitive (Be detection limit of 5 ~ 10 μg L^-1^) [10] but require extensive sample preparation, derivatization and relatively large sample volumes. Gas chromatographic methods have sufficient sensitivity and selectivity when coupled with mass spectrometry (Be detection limit of 30 ~ 40 μg L^-1^) [14], but they require a laborious, multi-step derivatization. Atomic emission, either arc emission or inductively coupled plasma emission are sensitive and accurate (Be detection limit of 0.2 ~ 1 μg L^-1^) [16], but often require large sample volumes and multi-steps preparation often including digestion. Inductively coupled plasma mass spectrometry (ICP-MS) recently became the methode of choice for biomonitoring trace elements in biological matrices (blood, plasma, urine, etc). It is a multi-elementary method that offer outstanding sensitivity, accuracy and detection limit in the low ng L^-1 ^with a reported beryllium detection limit in the range of 5 to 20 ng L^-1 ^depending on the matrix [20]. As for the atomic absorption methods, graphite furnace atomic spectrometry (GF-AAS) with a suitable chemical modifier (such as, lutetium [26], ammonium 12-molybdophosphate [27], palladium [28] and magnesium nitrate [24]) has been widely used for the analysis of beryllium in urine, environmental samples and tissue digests with detection limits in the range of 2 to 20 ng L^-1^. Limited work is published on the determination of beryllium in human serum, blood or biological fluids. The objective of this study is to develop a simple procedure for the direct determination of beryllium in undigested micro quantities of human blood and serum using GF-AAS as a routine analytical method. The analyses were performed by simple 5-fold and 8-fold dilution of the serum and blood respectively with a Nash reagent (NR) containing nitric acid, ammonium hydroxide, Triton X-100, antifoam B and EDTA [21,29]. We also compared three different experimental setups: using no modifier, using magnesium nitrate and using palladium/citric acid (reduced palladium) as chemical modifiers in order to study their effect on beryllium quantification in such complex matrices. We selected magnesium nitrate, known to improve beryllium sensitivity by a factor of 1.5 to 2 in matrices rich in halide ions (e.g. urine and environmental samples [24,27]) and reduced palladium, one of the commonly-used modifiers in GF-AAS analysis known to stabilize the atomization process regardless of the matrix complexity were evaluated [30-32]. Optimal pyrolysis and atomization conditions, detection limits and characteristic mass (the mass of element generating 0.0044 absorbance) were determined in order to develop a rapid and precise method for beryllium analysis in human blood and serum. The accuracy of the analytical method is tested with a control sample (Seronorm trace elements whole blood (STEWB) Level 2).

## Results and discussion

### Optimization of the furnace temperature program

**Figure 1:**
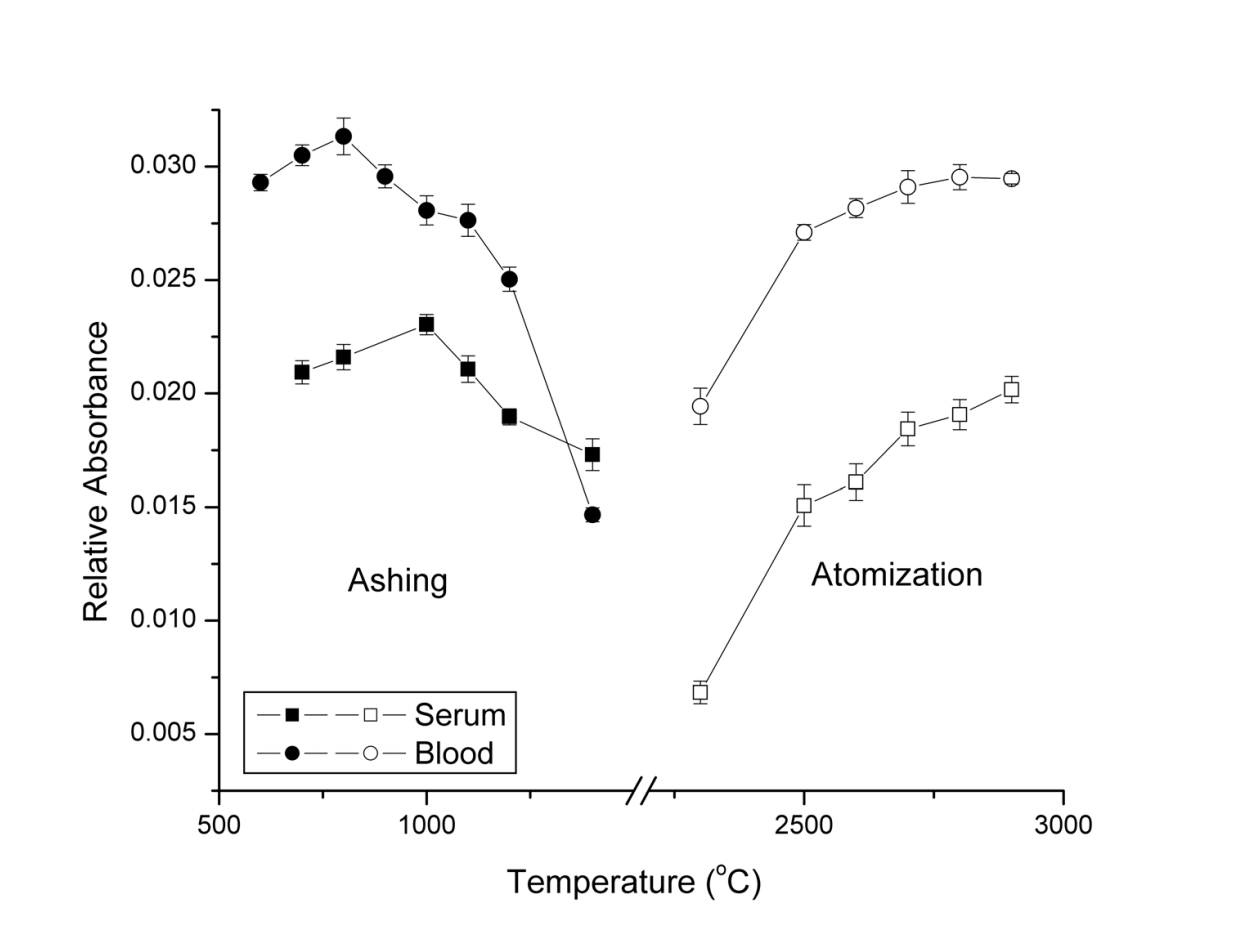
Thermal pre-treatment pyrolysis and atomization curves for 5-fold diluted non spiked serum and 8-fold diluted non spiked blood without the addition of any chemical modifiers.

Beryllium has a high boiling temperature of 2479°C. In general, it should not be lost during the pyrolysis stage unless it is complexed with organic compounds or halide ions. In all experimental set-ups, a pre-pyrolysis stage at 450°C was introduced in the temperature program to generate a slow pyrolysis of the blood and serum matrices. This pre-pyrolysis step was found necessary when using palladium as chemical modifier with citric acid as a reducing agent. It ensures the presence of reduced palladium at the very early stages of the temperature program [33,34]. It was also reported that the introduction of a pre-pyrolysis step in the temperature program helps the removal of smoke, especially when analysing matrices rich in organic matter [30]. In an attempt to optimize the temperature program for beryllium determination in serum, we first measured the relative absorbance of beryllium in a 5-fold diluted non spiked serum at a fixed atomization temperature (2900°C) while varying the pyrolysis temperature from 700 to 1400°C. We found that the optimal pyrolysis temperature generating the highest beryllium signal was 1000°C. We then fixed the pyrolysis temperature at its optimal temperature and varied the atomization temperatures between 2300 and 2900°C and found that the optimal atomization temperature was 2900°C. The background signal was decreased by 79% when pyrolysis temperature was increased from 800 to 1000°C. The same optimization work was reproduced for beryllium in an 8-fold diluted non spiked blood. The optimal pyrolysis temperature was found to be 800°C while the optimal atomization temperature was found at 2800°C. Background signal decreased by 48% when we ramped the pyrolysis temperature from 800 to 900°C but the beryllium absorbance also decreased by 10%. Figure 1 shows the complete pyrolysis and atomization diagram of beryllium in serum and blood.

### Palladium/citric acid as a chemical modifier

**Figure 2:**
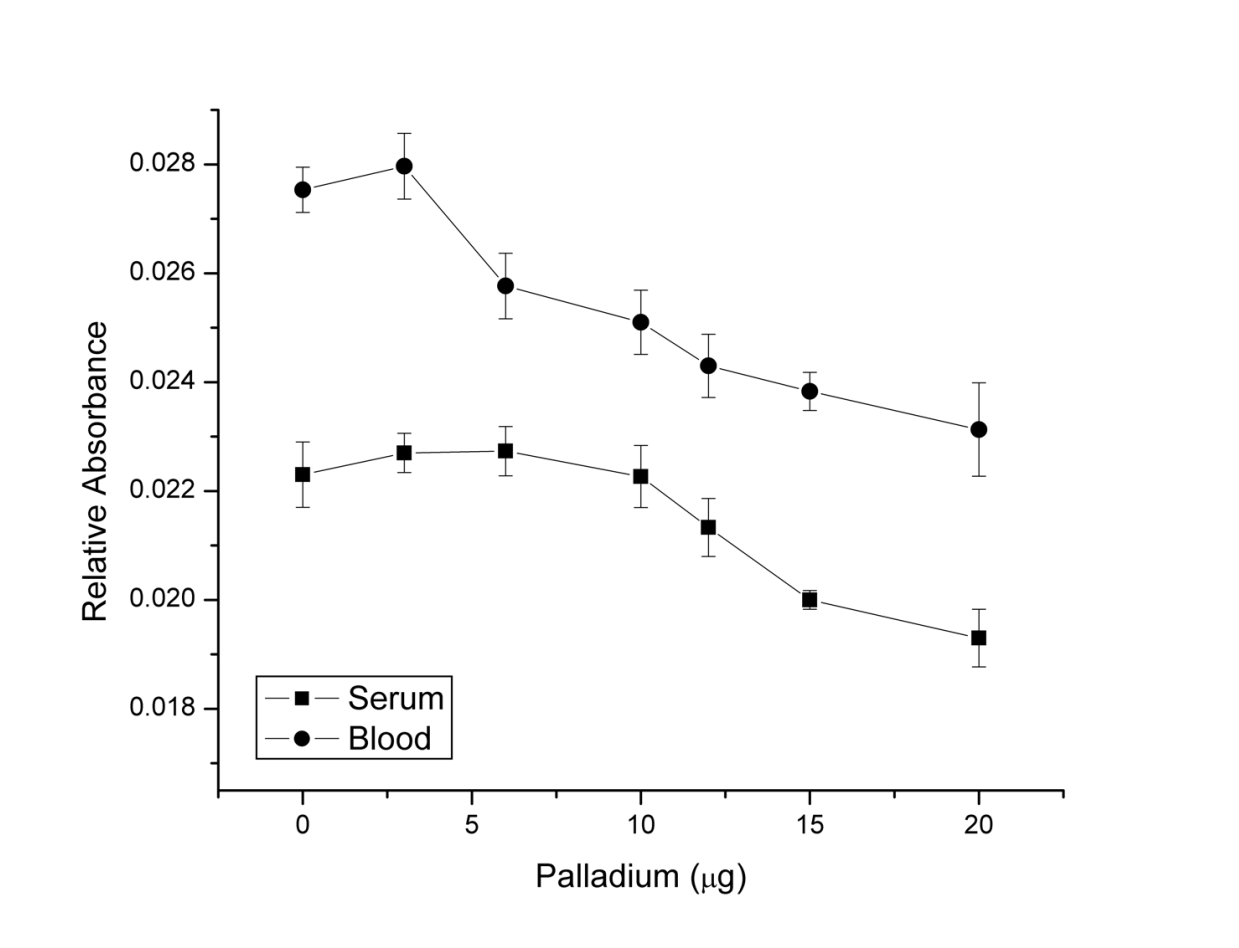
Influence of palladium mass on the relative absorbance of beryllium in 8-fold diluted blood and 5-fold diluted serum samples.

**Figure 3:**
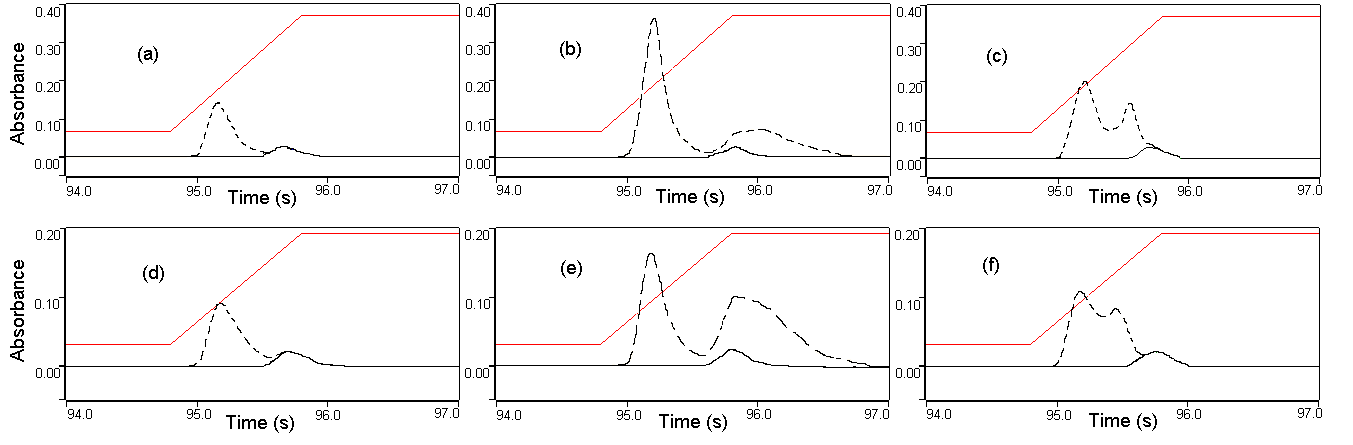
Variations in the background signal; (a) blood without modifier; (b) blood with 15 μg of Palladium; (c) blood with 10 μg Magnesium; (d) serum without modifier; (e) serum with 10 μg Palladium; (f) serum with 12 μg Magnesium.

Reduced palladium is an excellent modifier for GFAAS. It decreases matrix interferences, prevents elements vaporization during pyrolysis. It also generates a reproducible signal at high atomization temperatures due to the formation of Pd-metal compound [35-37]. We used citric acid as a reducing agent over ascorbic acid in order to eliminate excess injection. Ascorbic acid needs to be prepared and injected separately because it instantly reduces the palladium at room temperature while nitric acid only reduces palladium at higher temperatures [33]. At their respective optimal pyrolysis and atomization temperatures, 10 μL of the working palladium/citric acid solutions was co-injected with 20 μL of either 5-fold diluted serum or 8-fold diluted blood. For beryllium in serum, no statistically significant changes (one-sample T-test, p > 0.05) were noticed in the relative absorbance when 3 or 6 μg of palladium were introduced in the graphite furnace (an increase of 1.8 and 1.9% respectively). In contrast, the relative absorbance of beryllium continuously decreased when higher amounts of palladium were tested reaching a 14% reduction at 20 μg (Figure 2). The background signal increased dramatically by 130% when only 3 μg of palladium was used and continued to increase with increasing palladium mass introduced into the graphite furnace, reaching a maximum of 150%. We also noticed a change in the appearance of a background signal on top of the beryllium signal (Figure 3) that kept increasing linearly with the increasing mass of palladium injected in the graphite tube (y = 0.0091 x ; r^2 ^= 0.93). For beryllium in blood, no statistically significant changes were observed in the relative absorbance measured following the addition of 3 μg of palladium, yielding a 1.6% increase followed by a continuous decrease as the quantity of introduced palladium increased, up to an 8% reduction at 20 μg (Figure 2). The background signal of blood also increased with increasing palladium quantities and had a similar pattern of that found for beryllium in serum with the appearance of a background signal on top on the beryllium signal (Figure 3). The increase in the background signal varied from 133% at 3 μg, reaching a maximum of 165% for 20 μg of injected palladium (y = 0.0095 x ; r^2 ^= 0.94). Since the obtained background signal increased linearly with an increasing mass of injected palladium into the graphite tube, it is likely that this was due the atomization of the reduced palladium itself (boiling temperature of 2963°C). Because of the non-significant improvements in the relative absorbance and the increase in background signal, palladium/citric acid was not retained as a chemical modifier for beryllium in blood or serum.

### Magnesium as chemical modifier

**Figure 4:**
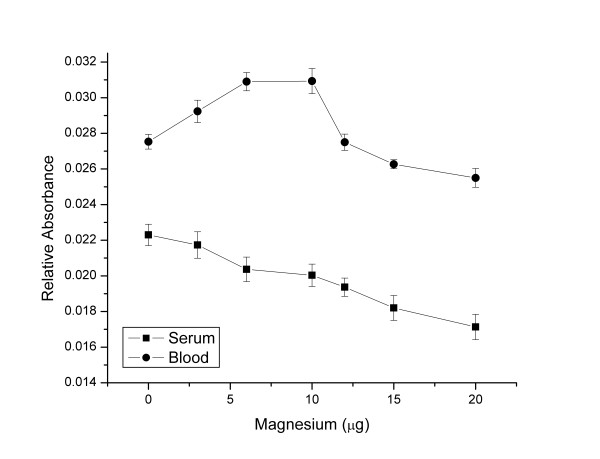
Influence of magnesium mass on the relative absorbance of beryllium in 8-fold diluted blood and 5-fold diluted serum samples.

The same experimental set-up was used to study the effect of magnesium on the relative absorbance of beryllium in 5-fold diluted serum and 8-fold diluted blood samples at their optimal pyrolysis and atomization temperatures. In serum, magnesium suppressed the relative absorbance of beryllium. This suppression increased with the increasing mass of magnesium introduced into the graphite furnace and varied from 2.5% at 3 μg to 23% at 20 μg (Figure 4). The background signal increased significantly by 50% when only 3 μg of Mg was introduced in the graphite furnace reaching a maximum of 80% when 20 μg of Mg was used as chemical modifier. We also noticed a change in the pattern of the background signal. A second background peak was noticed at around 2000°C (Figure 3), which increased linearly in intensity with increasing mass of magnesium injected into the graphite tube (y = 0.0071 x ; r^2 ^= 0.93). Such an observation confirms that the second background peak was caused by the atomization of magnesium. As for beryllium in blood, we found that the addition of magnesium nitrate as a chemical modifier tended to increase the relative absorbance of beryllium. This increase is significant (T-test, p < 0.05) and reached a maximum of 12% when 6 or 9 μg of magnesium were co-injected with the blood sample into the graphite furnace. The introduction of 3 μg also increased the relative intensity of beryllium by 6%. At higher magnesium concentrations, the relative absorbance of beryllium tended to decrease (Figure 4). The background pattern was similar to that found with serum, with the appearance of a second background peak at ~2000°C (Figure 3). The background intensity increased form 55% to 81% when we increased the concentration of introduced magnesium from 3 μg to 20 μg (y = 0.0074 x ; r^2 ^= 0.95). 6-μg amount of magnesium was retained as a chemical modifier for beryllium in blood despite the increase in the background signal because it appears earlier in the temperature program and does not interfere with the beryllium signal (Figure 3).

### Analysis of blood

Analysing undigested blood by GFAAS could be problematic due to the build-up of carbon residues in the graphite tube. Therefore, an 8-fold dilution of blood with the Nash reagent coupled with an optimal temperature program eliminated the deposition of carbon. Neither the precision nor the sensitivity was affected after at least 100 injections, as verified by the analysis of the reconstituted blood control material (STEWB level 2). We also performed a standard addition on the reconstituted blood control to validate the use of aqueous calibration and found no significant differences between both curves (y = 0.0692 x - 0.0019 ; r^2 ^= 0.99 for the aqueous calibration and y = 0.0682 - 0.0022; r^2 ^= 0.99 for the standard addition on the reconstituted blood control). Using the optimal temperature program for the 8-fold diluted blood and an aqueous beryllium standard with 6 μg of magnesium nitrate as chemical modifier, we measured the beryllium concentration in the blood of ten non-exposed individuals and found that it varied from 0.48 to 0.74 μg L^-1 ^with an average of 0.63 ± 0.08 μg L^-1 ^(± SD) (Table 1). We obtained a slightly lower mean beryllium concentration in blood than reported by [22], (0.94 ± 0.38 μg L^-1 ^(n = 10)). No significant differences were found between the two sets of data due to the large standard deviation reported in the previous study. Beryllium concentrations in blood of non-occupationally exposed individuals depend on a variety of factors such as smoking, eating and drinking habits, as those represent the main exposure routes for beryllium intake [38].

**Table 1:** Beryllium contents in blood and serum of ten individuals. Averages of triplicate measurements with standard deviations are given.

Sample	Sex	Smoker	Blood (μg L^-1^)	Serum (μg L^-1^)
1	F^†^	S^Ŧ^	0.71 ± 0.09	0.49 ± 0.08
2	F	NS^ŧ^	0.64 ± 0.09	0.43 ± 0.09
3	F	S	0.74 ± 0.06	0.45 ± 0.08
4	F	NS	0.67 ± 0.08	0.40 ± 0.08
5	F	NS	0.57 ± 0.06	0.46 ± 0.09
6	M	NS	0.68 ± 0.04	0.40 ± 0.05
7	F	NS	0.64 ± 0.08	0.41 ± 0.04
8	F	NS	0.67 ± 0.09	0.42 ± 0.04
9	F	NS	0.48 ± 0.06	0.43 ± 0.03
10	M^‡^	NS	0.55 ± 0.07	0.42 ± 0.06
	Mean		0.63 ± 0.08	0.43 ± 0.03

^† ^Female; ^‡ ^Male; ^Ŧ ^Smoker; ^ŧ ^non smoker.

The method detection limit was determined as the concentration corresponding to three times the standard deviation of 8 replicates of the lowest standard (0.05 μg L^-1^). We found a beryllium detection limit of 7 ng L^-1 ^in blood. The beryllium characteristic mass was found to be 0.20 (± 0.02) pg, of the same order of magnitude as that reported by the manufacturer: 0.5 pg determined in 0.1% nitric acid matrix. It was calculated as the mass of beryllium in 8-fold diluted blood that yields an absorbance equal to 0.0044 (1% absorption) in the peak height mode, with a new pyrolytic coated graphite tube and under the optimal furnace temperature program [39]. This similarity in the beryllium characteristic mass reassures us that our experimental conditions have eliminated signal suppression for the blood matrix. The accuracy of the method was verified by analysing the STEWB level 2 control sample with a beryllium concentration of 5.9 (± 0.5) μg L^-1^. Accuracy values of four different control samples prepared by 10 or 20-fold dilution of STEWB level 2 with NR were found to be 100 (± 1) %. For the actual 10 samples we tested, neither sex nor smoking habit had a statistically significant influence on the concentration of beryllium in blood (T-test, p > 0.05), but for the two smokers, data points were higher. A larger dataset would be necessary to properly explore exposure questions and may yield interesting differences.

### Analysis of serum

We found that beryllium concentrations in the serum of ten non-exposed individuals, analysed under optimal temperature, using aqueous beryllium standards without any chemical modification, varied from 0.40 to 0.49 μg L^-1 ^with an average of 0.43 ± 0.03 μg L^-1 ^(± SD) (Table 1). The beryllium detection limit was found to be 2 ng L^-1 ^determined as the concentration corresponding to three times the standard deviation of 12 replicates of the lowest calibration standard (0.05 μg L^-1^). The beryllium characteristic mass in 5-fold diluted serum was found to be 0.22 (± 0.07) pg, slightly lower than that reported by the manufacturer: 0.5 pg determined in 0.1% nitric acid matrix [39]. This also confirms that no signal suppression was caused by the serum matrix under our experimental conditions. The accuracy of the method was verified using the same control material (STEWB level 2). We obtained an accuracy of 97.5 (± 2.5)% on four different control samples. As observed earlier for blood, no significant influence of beryllium concentration in serum is attributed to sex or smoking (T-test, p > 0.05).

## Conclusion

We found a significant difference between the beryllium concentration in blood and serum (p < 0.05). We measured higher beryllium concentration in blood than in serum. On average, we noted a 30 ± 10% increase in the blood beryllium concentration over serum. These findings suggest that roughly two thirds of the beryllium concentration in the blood stream is found in the serum and only one third is attached to the blood clot. Despite the complexity of serum and blood matrices, no signal suppression was noticed following our optimal conditions and our beryllium detection limits (2 to 7 ng L^-1^) using the Varian AA280Z Zeeman atomic absorption spectrometer are among the lowest reported to date. [40] and [26], reported a detection limit of 370 ng L^-1 ^and 4.3 ng L^-1 ^respectively for beryllium in urine by graphite furnace atomic absorption spectrometry. In a similar work, [27], and [24], reported a detection limit of 50 ng L^-1 ^for beryllium in urine. In water, [25] found 2.3 ng L^-1 ^while [41], found 10 ng L^-1^. The proposed method is ideally suited to evaluate occupational exposure and other factors contributing to beryllium risks.

## Experimental

### Instrumentation

A Varian AA280Z Zeeman atomic absorption spectrometer, equipped with a Zeeman background corrector, a GTA 120 graphite tube atomizer and a PSD 120 programmable sample dispenser was used for the atomic absorption measurement of beryllium at 234.9 nm with a spectral band width of 1.0 nm. A beryllium hollow cathode lamp (Varian, Part No. 5610100500) was used as a light source operated at 5 mA. Pyrolytic graphite-coated partitioned tubes (Varian partition tubes, Part No. 63-100012-00) were used in all experiments. High purity argon (99.99%) was used as the carrier gas. Instrument control, sample results, signal graphics and data collection are controlled by the SpectrAA Worksheet software for Windows^® ^XP operating system (Version 5.1, Varian, Australia 1997–2006). Peak areas were used for the optimisation of the furnace program, the choice of the appropriate experimental setup and the quantification of beryllium in blood and serum. The furnace programs for serum and blood analysis are listed in Table 2.

**Table 2:** Instrument conditions and furnace programme for the determination of beryllium (argon flow rate was set at 0.3 L min-1, except during atomization)

Step	Temp (°C)	Time (s)
Drying	85	5
Drying	95	30
Drying	120	20
Pre-pyrolysis	450	22
Pyrolysis	1000^†^; 800^‡^	17
Atomizing	2900^†^; 2800^‡^	3
Cleaning	2900	2

^† ^Serum analysis

^‡ ^Blood analysis

### Reagents and solutions

All reagents are of analytical grade, unless otherwise stated. Antifoam B silicone emulsion (J.T. Baker, NJ, USA), ammonium hydroxide (certified A.C.S. Plus, Fisher scientific, NJ, USA), beryllium standard solution (Specpure, Alfa Aesar, MA, USA), palladium matrix modifier (Sigma-Aldrich, USA), ethylenediaminetetraacetic acid disodium salt dihydrate (EDTA) (Fluka Chemika, Switzerland), nitric acid (trace metal grade, Fisher Scientific, Ontario, Canada), Triton X-100 (Acros, NJ, USA), citric acid and magnesium nitrate were purchased from Fisher-scientific (Ottawa, Ont, Canada). Seronorm trace elements whole blood (STEWB) Level 2 (Ref # 201605; Lot # 0503109) was purchased from SERO (Billingstad, Norway).

Blood and serum samples were diluted with a Nash Reagent (NR) prepared weekly containing 5% (v/v) nitric acid, 5% (v/v) of ammonium hydroxide, 0.2% (v/v) Triton X-100, 0.2% (v/v) antifoam B and 0.5% (w/v) of EDTA. A 50 μg L^-1 ^working beryllium(II) solution was prepared by dilution of the beryllium stock solution (1000 μg L^-1^) in 2% (v/v) HNO_3_. Beryllium standard solution was prepared daily by dilution of the working beryllium(II) solution with the Nash Reagent to give a final concentration of 0.5 μg L^-1^. A series of palladium working solutions containing 300, 600, 1000, 1200, 1500 and 2000 mg L^-1 ^were prepared by dilution of the stock solution (10 000 mg L^-1^) in 2% (v/v) HNO_3 _and 2% (w/v) citric acid aqueous solutions. Another series containing 300, 600, 1000, 1200, 1500 and 2000 mg L^-1 ^magnesium nitrate was prepared by dissolving the appropriate mass in 2% (v/v) HNO_3 _aqueous solution. A 10-μL volume of the chemical modifier or Nash Reagent (when no modifier is used) was co-injected with 20-μL of the sample into the furnace.

### Blood and serum Samples

Blood and serum samples of 10 individuals (8 females and 2 males) were collected respectively in BD Vacutaine (sodium Heparin) and BD Vacutaine (SST) respectively (BD Franklin lakes (NJ, USA)). All subjects live in the region of Montreal, Quebec, Canada with no previous history of occupational exposure to beryllium, from questionnaire-based interviews. Blood was diluted 8-fold and serum 5-fold with the Nash Reagent. Triplicates of blood and serum samples were refrigerated at 4°C until analysis. All samples were analysed on the day of collection.

## Authors' contributions

All authors contributed to the experimental design. CHS carried out nearly all of the laboratory work, the initial interpretation of the data and the initial write-up. PB and MF contributed their expertise especially with the biological work, and SS focused on the analytical chemistry.
